# Investigating Explanatory Factors of Machine Learning Models for Plant Classification

**DOI:** 10.3390/plants10122674

**Published:** 2021-12-05

**Authors:** Wilfried Wöber, Lars Mehnen, Peter Sykacek, Harald Meimberg

**Affiliations:** 1 Department of Integrative Biology and Biodiversity Research, Institute of Integrative Conservation Research, University of Natural Resources and Life Sciences, Gregor Mendel Str. 33, 1080 Vienna, Austria; meimberg@boku.ac.at; 2 Department Industrial Engineering, University of Applied Sciences Technikum Wien, Höchstädtplatz 6, 1200 Vienna, Austria; 3 Department Computer Science, University of Applied Sciences Technikum Wien, Höchstädtplatz 6, 1200 Vienna, Austria; mehnen@technikum-wien.at; 4 Department of Biotechnology, Institute of Computational Biology, University of Natural Resources and Life Sciences, Muthgasse 18, 1190 Vienna, Austria; sykacek@boku.ac.at

**Keywords:** deep learning, machine learning, plant leaf morphometrics, explainable AI

## Abstract

Recent progress in machine learning and deep learning has enabled the implementation of plant and crop detection using systematic inspection of the leaf shapes and other morphological characters for identification systems for precision farming. However, the models used for this approach tend to become black-box models, in the sense that it is difficult to trace characters that are the base for the classification. The interpretability is therefore limited and the explanatory factors may not be based on reasonable visible characters. We investigate the explanatory factors of recent machine learning and deep learning models for plant classification tasks. Based on a Daucus carota and a Beta vulgaris image data set, we implement plant classification models and compare those models by their predictive performance as well as explainability. For comparison we implemented a feed forward convolutional neuronal network as a default model. To evaluate the performance, we trained an unsupervised Bayesian Gaussian process latent variable model as well as a convolutional autoencoder for feature extraction and rely on a support vector machine for classification. The explanatory factors of all models were extracted and analyzed. The experiments show, that feed forward convolutional neuronal networks (98.24% and 96.10% mean accuracy) outperforms the Bayesian Gaussian process latent variable pipeline (92.08% and 94.31% mean accuracy) as well as the convolutional autoenceoder pipeline (92.38% and 93.28% mean accuracy) based approaches in terms of classification accuracy, even though not significant for Beta vulgaris images. Additionally, we found that the neuronal network used biological uninterpretable image regions for the plant classification task. In contrast to that, the unsupervised learning models rely on explainable visual characters. We conclude that supervised convolutional neuronal networks must be used carefully to ensure biological interpretability. We recommend unsupervised machine learning, careful feature investigation, and statistical feature analysis for biological applications.

## 1. Introduction

Precision farming and plant science benefits from the recent trend of digitalization and robot-based automation. A vast amount of data is accessible in databases [1,2,3,4], which enable novel machine learning based methodologies for visual plant or crop inspection and identification. Convolutional neuronal networks (CNNs) achieved the highest precision in plant identification tasks as shown in the meta analysis [5]. A CNN is an artificial neuronal network based architecture including convolutional layers [6]. CNNs were used for plant identification in [7]. Different CNN architectures relying on Googlenet [8] were proposed to identify 100 ornamental plant species [9]. Existing CNN architectures [10,11] were extended to identify crops and weeds [4]. Another approach is a robot-based plant identification system for agriculture that had been implemented using two CNNs for pixel-wise classification of crops [12]. In the same context, novel architectures for real time weed and plant identification were used in [13]. CNNs can further be used to identify plant diseases [14,15,16,17].

The studies mentioned above are heavily based on CNNs. These models implement the classification task by learning from example images. Nevertheless, the studies report technical metrics such as the prediction accuracy but do not analyze the explanatory factors for the CNN decisions. The majority of plant identification studies is done by computer vision experts and a lack in interdisciplinary work [18]. In the recent plant science studies [19,20], the authors showed that explanatory factor extraction can be used for reliability investigation. Nevertheless, recent progress in the machine learning community showed, that CNNs may use image regions that happened to be related to the classification goal without any reasonable context, the so-called ”clever-Hans phenomenon” [21]. This phenomenon is critical for both scientific analysis and reliable application implementation. It has been argued, that any artificial intelligence must understand the fundamental explanatory factors used for decisions [22]. In order to explain the decision of deep learning models, the machine learning community proposed strategies [23,24,25] as well as methods such as the layer-wise relevance propagation [25,26]. Those methods mainly explain decisions of CNNs or gather these decisions for large databases [27]. Nevertheless, the explanatory factors coded in the models remains invisible in the models.

However, ref [28] points out that unsupervised learning may overcome recent problems of supervised CNNs. In contrast to supervised learning, unsupervised learning tackles the identification of explanatory factors. Based on these explanatory factors, tasks such as classification can be implemented. Ref [29] investigated the behavior of a CNN for a Nile tilapia classification task in comparison to a model that has been trained unsupervised. They analyzed CNN decisions and found that it outperforms other machine learning models in terms of prediction accuracy but used background image regions (e.g., the mounting pin of the specimens). The unsupervised model was found to be inferior in terms of classification performance but robust in terms of biological interpretability. The authors found explanatory factors in the trained model and overcame the problem of model explanation relying on individual predictions. However, this interpretability was achieved by manual feature selection.

**Figure 1:**
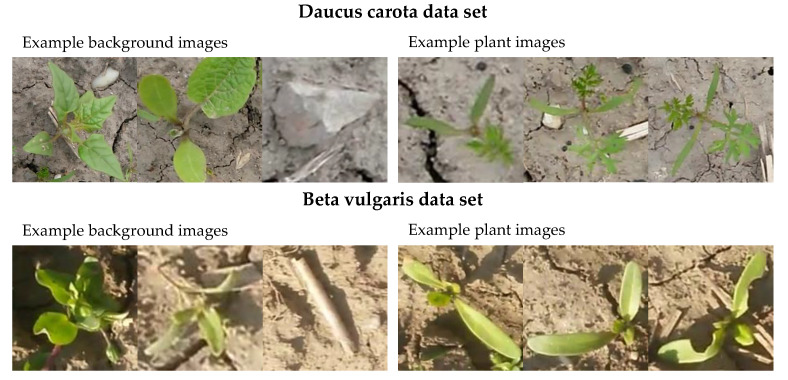
Randomly chosen example images of both used datasets. The left side shows three background images (soil, other plants, etc.) and the right images show example images of the plant to classify.

In plant identification this might play a larger role, because images can contain diverse background and models based on supervised CNNs can draw conclusions based on biological uninformative image regions. To overcome the aforementioned problems, we propose unsupervised learning based models as an alternative to supervised CNNs for plant classification, where the model decision must be explained relying on individual predictions. Our pipeline extends the framework of [29] by automating the feature selection procedure. We use Spearman’s rank correlation test [30] to identify significant explanatory factors and use only these factors for classification. The information content is evaluated based on two plant classification data sets, where we compare supervised CNNs to unsupervised learning based methodologies. The data used for the experiments is based on two image data sets generated during field experiments. The former data set shows images of *Daucus carota* and the latter data set show images of *Beta vulgaris*. In both data sets, additional images showing soil, stones, and other plants do exist. We chose these data sets due to different leaf shape complexity and leaf architecture. Example images of both data sets are shown in Figure 1.

All experiments rely on pre-processed RGB images. To this end, we follow the recommendation of [13] and converted all images using the excess green index.

This study evaluates the classification performance of plant recognition models. Due to the fact that many plant detection approaches are heavily based on classification models (e.g., [31]) we currently do not evaluate detectors such as [32]. We implement the machine learning models in order to identify the plants in images. We compare the supervised Conv-net D (VGG16) CNN [33] including decision explanation with two two-staged unsupervised learning based approaches. In the latter, the first stage tackles the extraction of reasonable features (e.g., explanatory factors) using unsupervised models, and the second stage implements the classification task. In the first stage, we use a Bayesian Gaussian process latent variable model (B-GP-LVM) [34] previously used for Nile tilapia analysis [29]. To compare the B-GP-LVM to a recent deep learning-based model, we additionally implement a convolutional autoencoder (cAE) [35]. We apply the explanation pipeline for the Bayesian Gaussian process latent variable model of [29] in this study and extend this pipeline for cAE. In the second stage of our pipeline we use a support vector machine (SVM) [36] to classify the extracted features. We compare the results using the following steps:

- Biological interpretation: We interpret the visual reasons for classification of the used supervised CNNs using layer-wise relevance propagation (LRP) [25,26] and for the unsupervised models the sampling based methodology introduced in [29,37]. We evaluate the significance of the pixels in the resulting saliency maps (heatmaps) using the pixel-wise significance test introduced in [29]. We refer this test as the *test for saliency maps*. We extend the pipeline of [29] using statistical feature analysis instead of manual selection.
- Classification accuracy: We compare the predictive accuracy using a five-fold cross-validation in 10 iterations relying on reshuffled data sets.
- Statistical analysis: Additional to the predictive accuracy, we compare the model’s uncertainty using the mutual information [38] to get more details about the prediction procedure. Finally, we compare the error rate of the two-staged approaches to the CNN using McNemar’s test [30].

**Figure 2:**
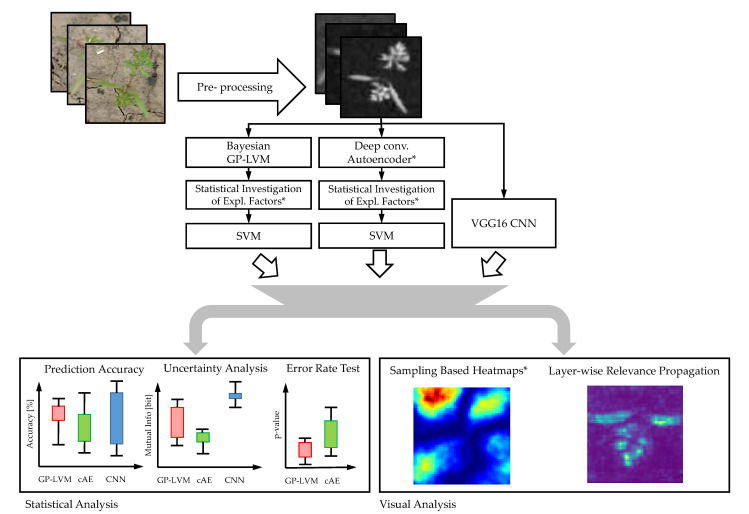
Data processing pipeline used in this study. The classification task is implemented using three different approaches. The results are analyzed and compared. The objective of our study is the evaluation of the explanatory factors of machine learning models. To this end, we compare the VGG16 CNN [33] to a Bayesian GP-LVM [39] and deep convolutional autoencoder [35] based classification methodology. We compare the models using statistical analysis metrics relying on the prediction accuracy, mutual information, and McNemars test for error rates. Finally, the explanatory factors are visualized using layer-wise relevance propagation [26] and the sampling method of [29,37]. The contribution of this study is marked with an asterisk. The sampling based heatmaps for B-GP-LVM was used to extract the explanatory factors of the cAE.

The remaining part of this study presents the results of our model comparison based on the two data sets. We used the data processing pipeline shown in Figure 2.

In this pipeline, we initially learn features using the B-GP-LVM and a deep convolutional autoencoder followed by explanation factor extraction and statistical investigation in order to select features for processing. Our contribution and extension of existing pipelines are marked with an asterisk. The selected features are used for classification using the SVM. In the VGG16 CNN, a single stage learns features and implements the classification task. The approaches are based on the same training/test split. For each experiment, the predicted labels and prediction probabilities are stored. We compare the model performance using the aforementioned metrics. Afterwards we analyze the explanatory factors of all used models. Finally, we conclude this study by considering the predictive performance in contrast to the explanatory factors unveiled during the model analysis.

The main contribution of this study is the detailed model analysis of a plant classification task. This includes the visualization of the explanatory factors. Furthermore, we extend the pipeline previously provided by [29] relying on statistical feature selection and apply the new pipeline for convolutional autoencoders. All used images as well as the processing pipeline including the software is available under https://github.com/TW-Robotics/Plant_Unsupervised (released on 3 December 2021).

This study is structured as follows. Section 2 discusses the state of the art of explainable artificial intelligence for machine learning based computer vision applications. Afterwards, we present the classification results and explanatory factor extraction. Section 4 discusses the results. Finally, Section 5 describes the used materials before we summarize this study in Section 6.

## 2. Related Work

Linear models in statistics such as linear regression or logistic regression do have the advantage of being white-box models, where the explanatory factors can easily be investigated [40]. Similarly, linear models such as the Eigenfaces [41] relying on the principal component analysis (PCA) were used in computer vision. These models can be explained in a similar manner as linear regression models, but they suffer from a strong linear assumption. However, this interpretability is not available for (deep) machine learning models for image analysis.

The explanation of (deep) machine learning models describes the investigation of a trained model’s decision in an interpretable domain [25], such as heatmaps. This investigation is typically done after model training [24,25] and tackles the identification of reasons for individual prediction steps. These reasons are typically coded in the model, are not visible for the user and need to be investigated in order to verify the black-box models [42]. Several strategies exists to visualize explanations from the models, namely the investigation of the learned representation, the explanation of individual predictions, explaining the model behavior, and investigating explanatory factors relying on representative examples [42].

The investigation of those representative training examples was done in [43] in order to interpret the classifier. Similarly, [44] calculated representative examples to investigate the learned representations. However, these examples may not be helpful to interpret the decision of real images [24]. To overcome this limitation, [21,25,26] proposed the layer-wise relevance propagation and [45] proposed GradCAM as alternative strategies to visualize the reasons for individual CNN predictions. These methods can be extended for explaining multiple decisions and were used for investigating the clever-Hans phenomenon [27,46].

However, the aforementioned literature tackles the problem of explaining individual decisions from highly successful deep learning models. As previously discussed in [22], the latent explanatory factors need to be investigated in order to create robust and reliable systems. The explanation strategies discussed above mainly use individual decisions or representative samples to gather information of the latent explanatory factors. The explanatory factors coded in the deep learning models remain invisible. To overcome this problem, [28] proposed unsupervised learning. Inspired by the Eigenfaces, [29] proposed a method relying on the non-linear and non-parametric unsupervised Bayesian Gaussian process latent variable model [39]. The authors were able to overcome the clever-Hans phenomenon and provided a methodology, where the explanatory factors of the model are visualized and manually investigated. The explanatory factor analysis relies on the investigation of the trained models behavior and visualizes image regions used for feature extraction [29,37].

In this study, we extend the proposed method of [29] by using statistical investigation instead of manual feature selection. Furthermore, we applied their explanation pipeline for convolutional autoencoders. Table 1 summarizes the explanation strategies and show our contribution. As a supervised deep learning counterpart, we explain the decision of the VGG16 network using LRP and visually investigate the explanation of each sample.

## 3. Results

Before we present our results, we initially discuss the model selection procedure. Afterwards, the prediction performance is presented.

### 3.1. Model Selection

The hyperparameters for the B-GP-LVM as well as the cAE needs to be optimized. The kernel hyperparamesters and number of latent features were optimized for the B-GP-LVM. For the cAE, we optimized for code size.

**Figure 3:**
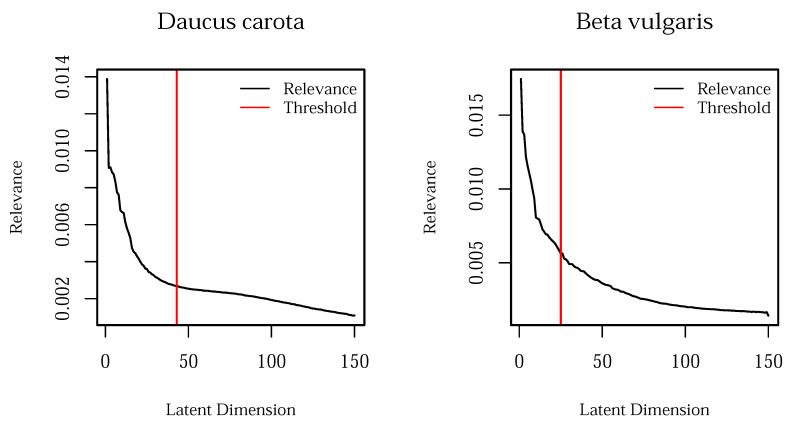
Relevance analysis of both data sets. The relevance threshold was determined by the median of the relevance increase.

The B-GL-LVM model selection relies on a step-wise procedure initially proposed in [29]. In this procedure, we maximize the log marginal likelihood. This optimization results in the maximum value for d=150 as well as 150 auxiliary points. We assume that the high variability occurs due to soil and other plants in the image data set. To reduce the number of usable features, we additionally analyze the kernel’s inverted lengthscale (relevance). The relevance based selection for both data sets is shown in Figure 3.

Relying on the median of relevance increase of each feature, we found a threshold of 43 features for *Daucus carota* and 25 features for *Beta vulgaris*. The remaining features were investigated relying on Spearman’s rank correlation test. This test analyzes the relation between the features and label. Features with p>0.1 were selected for further investigation. This procedure results in 14 features for *Daucus carota* and 11 features for *Beta vulgaris*.

**Figure 4:**
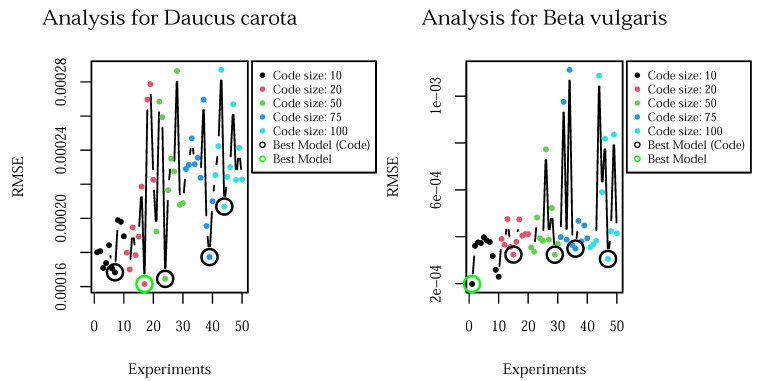
Analysis of the code size for cAE for both data sets. The model with lowest MSE is visualized with a green circle. The best model for each code size is shown in a black circle. The influence of the additional parameters can be seen in the high variation of the loss for larger code sizes.

The cAE optimization is based on the mean squared error (MSE). We analyzed the MSE for the code sizes {10,20,50,75,100} in 10 iterations. The result of our analysis is visualized in Figure 4.

This optimization results in 20 features for *Daucus carota* and 10 features for *Beta vulgaris*. Similarly to the B-GP-LVM features selection relying on Spearman’s test, the statistical test for the cAE results in 13 features for *Daucus carota* and 7 features for *Beta vulgaris*. To conclude this subsection we emphasize on the influence of the number of parameters in the model, which introduce high variation in the loss for larger code sizes.

### 3.2. Model Performance

**Figure 5:**
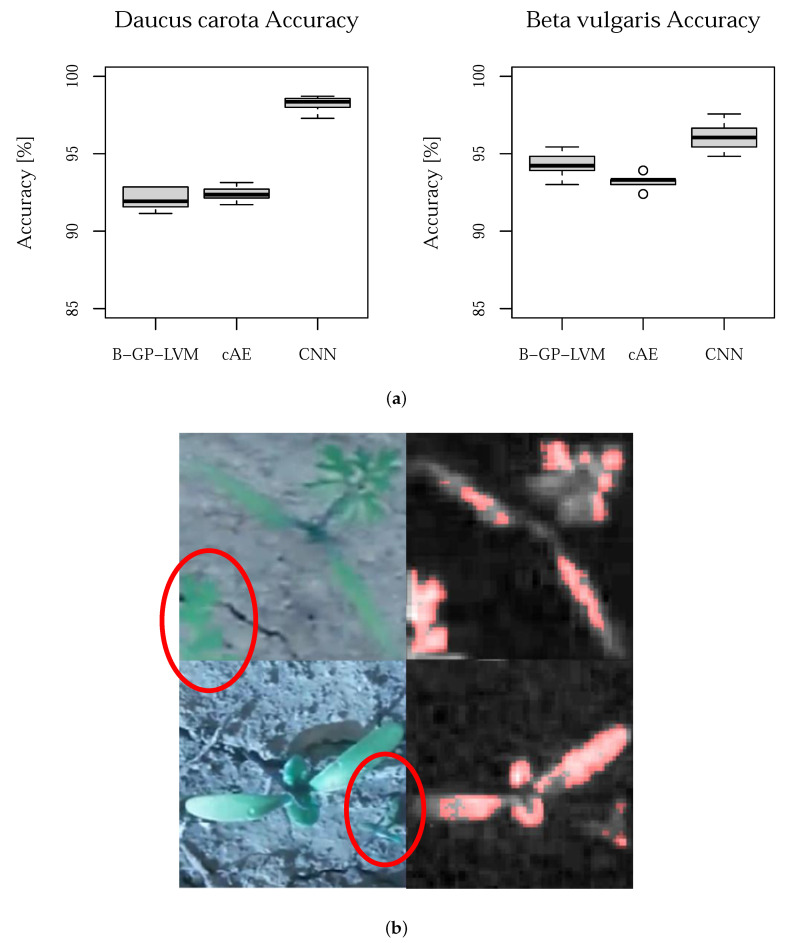
Prediction accuracy of the unsupervised models (**a**) Prediction accuracy of the proposed models in comparison to the CNN for *Daucus carota* (**left**) and *Beta vulgaris* (**right**). In both cases, the CNN outperforms the unsupervised models. In (**b**) Input image for CNN classification (**left**) and pixels used for decision (**right**), correctly classified images are shown. The CNN relies on the red colored pixels. These areas includes non-plant pixels.

The selected features are processed using a SVM including hyperparameter optimization relying on global hyperparameter optimization [47]. The experiments are based on 10 five-fold cross validation iterations relying on reshuffled data sets. The prediction accuracy (ACC) boxplot of all experiments including the CNN is shown in Figure 5. The mean accuracy for *Daucus carota* is 92.08% (B-GP-LVM), 92.38% (cAE) and 98.24% (CNN). Furthermore, the mean accuracy for *Beta vulgaris* is 94.31% (B-GP-LVM), 93.28% (cAE) and 96.10% (CNN). The left plot show the results for the *Daucus carota* data set and the right plot shows the result for the *Beta vulgaris* data set. However, we found biological uninterpretable reasons for the CNN decision. These uninterpretable regions may be the reason for the significant classification difference of the CNN. Examples are shown in the red circle on the right side of Figure 5.

**Figure 6:**
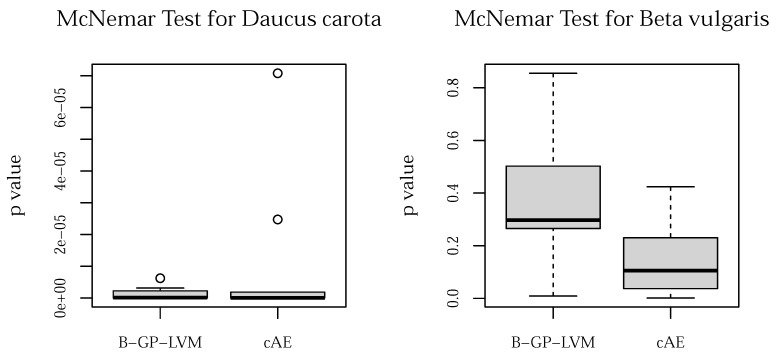
McNemar test for *Daucus carota* (**left**) and *Beta vulgaris* (**right**). Assuming α=0.05, we see that for *Beta vulgaris* the CNN error rate does not differ significantly from the proposed unsupervised models. However, we observed a significant error rate difference for the *Daucus carota* data set.

The McNemar test was used to evaluate the prediction performance differences of the proposed unsupervised models to the CNN. The results for both data sets are shown in Figure 6.

Assuming α=0.05, we can reject $H_{0}:$ *the error rate for both models are equal* for *Beta vulgaris* but not for *Daucus carota*.

**Figure 7:**
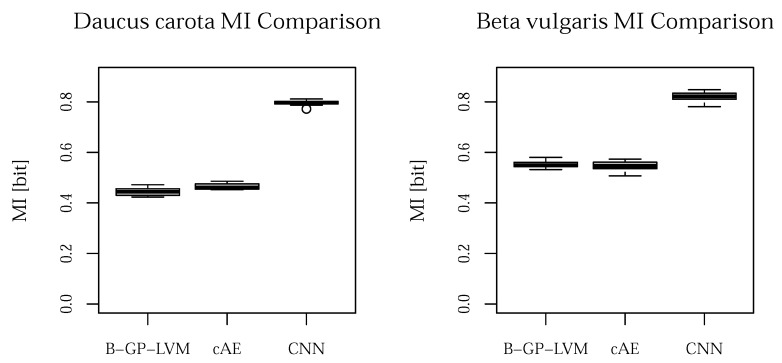
Mutual information of the performed experiments. For both data sets, the CNN outperforms the proposed models.

Finally, we analyze the models uncertainty using the mutual information (MI) [48]. Additionally to the ACC, the MI provides more details in the classification procedure considering the classification uncertainty. Classification models with an accuracy above 50% result in a MI value greater than zero. The MI for both data sets is visualize in Figure 7.

The MI plot shows that the CNN outperforms the other models in terms of low prediction uncertainty.

Based on the metrics above, the classifiers can be compared statistically. Nevertheless, as previously discussed in [21,29] these metrics are unable to quantify classification based on artifacts that happen to be related with the image label. To investigate the image regions used for the classification, we visualize these image regions using saliency maps. We apply a statistical test for saliency maps [29] to evaluate the pixel-wise significance.

**Figure 8:**
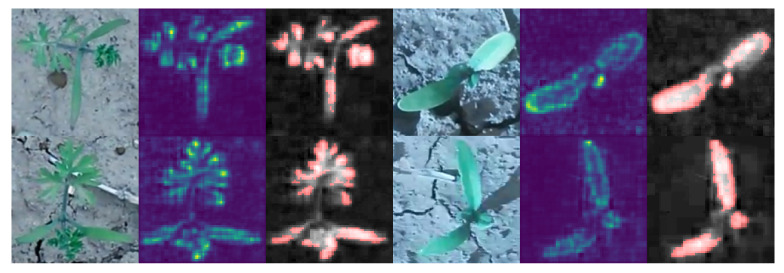
Layer-wise relevance propagation result for both data sets. For each example, the original RGB image (**left**), the LRP result (**center**) and the result of the statistical test for the saliency maps (**right**) is visualized. The results show that the CNN was able to focus on the plant leaf shape and architecture.

The CNN tries to learn useful patterns implicitly, in order to solve the classification task. However, this implicit plant representation cannot be evaluated directly, therefore the decision of the CNN, and the reasons for this decision must be evaluated per sample. For this purpose, we applied LRP [25,26] as well as a statistical test for saliency maps for the best iteration and fold in the experiments. The model decision for all plant images in the selected folds were analyzed. Examples are shown in Figure 8. The figure shows two randomly chosen examples of *Daucus carota* (left) and two examples of *Beta vulgaris* (right). The first column for each example shows the original RGB image, the second column shows the LRP result and the third column shows the result of the statistical test for saliency maps applied on the pre-processed excess green image.

The results show that the CNN focuses on the plant leaves and architecture for plant classification.

**Figure 9:**
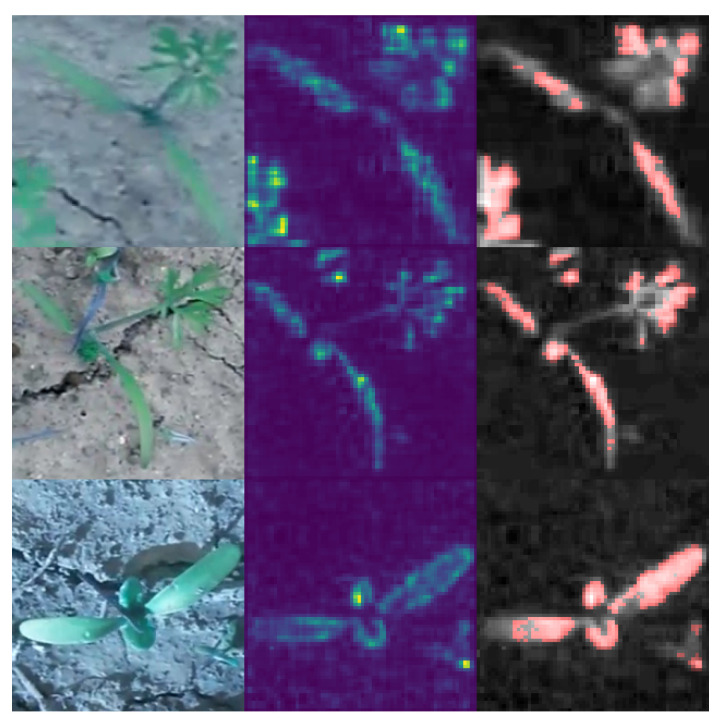
Example images showing correctly classified plant images, where parts of other plants were used for classification. The left images show the raw RGB images. The center images show the LRP result. The right images show excess green images (gray-scaled images) overlayed by the LRP results thresholded using the statistical test for saliency maps (red pixels).

Nevertheless, in the classification result of the best fold in the best iteration, we found 2 out of 66 examples for *Daucus carota* and 1 out of 46 for *Beta vulgaris* where the LRP shows biological uninterpretable image regions. For the purpose of this study the number of faulty samples is low. Nevertheless, detected faulty reasons for classification indicates faulty plant descriptions coded in the CNN architecture. The faulty examples are shown in Figure 9.

These results show that pixels of other plants are used to classify the images correctly. This indicates that these image regions were used to achieve the high prediction accuracy during training. Furthermore, the results show that the classification accuracy of the CNN is based on image regions that happen to relate with the image label.

**Figure 10:**
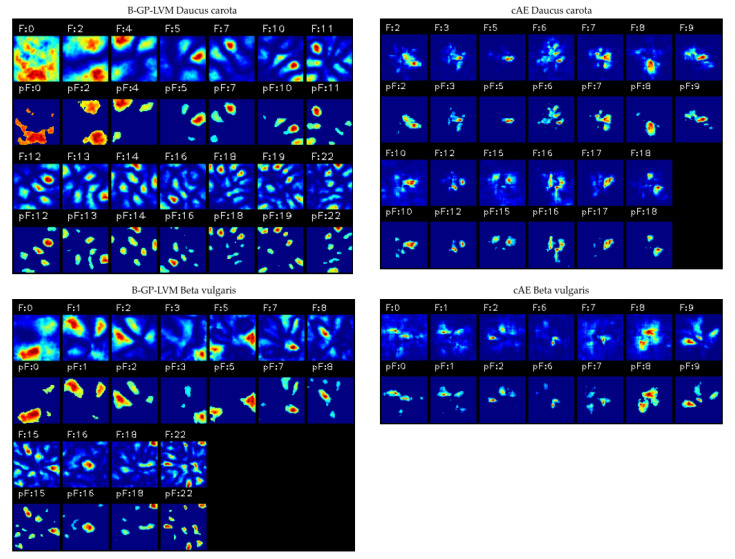
The learned features after feature selection for both data sets and unsupervised models. The images show the extracted features including the feature identification number (e.g., F:0) and the thresholded features using the statistical test’s *p* value (e.g., pF:0). Both models are able to extract leaf-like structures. The B-GP-LVM was able to extract leaf patches and background such as soil (e.g., F:4 for *Daucus carota*). The cAE is able to identify more complex leaf coherence but minor background information.

The proposed methodologies based on unsupervised learning tries to overcome this phenomenon by using statistical analysis for feature extraction. In the first stage, we extract image regions with high variability and selected features using statistical analysis. The feature visualization is based on [29,37], where we create saliency maps by sampling from the feature space and analyzing the resulting variance in the image space. Figure 10 shows the visualization of the learned features after feature selection based on a Spearman’s rank correlation test.

The models were able to extract parts of the leaf structure as well as the leaf architecture for both data sets. The results show that the cAE extracted less noisy image regions with fewer leaf area. The B-GP-LVM focused for both data sets on large image areas (e.g., F:0 and F:2). This indicates that the model selected those regions due to the lesser plant-based information in the image. Thus these regions are based on soil regions with high variability.

Different to CNNs, where the explanatory factors are coded in the model and cannot be visualized directly, we were able to visualize all explanatory factors for the unsupervised models visualized in Figure 10. Those features were used for classification. Nevertheless, the unsupervised model based feature visualization extracts image regions for both classes (plant and background). This is different to the CNN visualization, where pixels used for plant classification were highlighted. On the one hand, our analysis shows that the unsupervised models learn both plant and background features. On the other hand we can be sure that the classification is based on those visual characters.

## 4. Discussion

Our results show that the CNN outperforms the proposed unsupervised learning based methodologies in terms of prediction accuracy and uncertainty. Nevertheless, the CNN prediction performance difference was not significant for the *Beta vulgaris* data set. For the *Daucus carota* image data set the prediction error rate difference was significant. We assume that the complex leaf structure of *Daucus carota* and the optimized CNN architecture for images is the reason for the significant difference. Unfortunately, we found that the CNN used image regions that cannot be interpreted by biological means to achieve the higher accuracy.

Similarly to earlier findings for animal images [29], we conclude, that the higher CNN prediction accuracy may be based on clever-Hans predictors [21]. In that case the excellent CNN prediction accuracy is based on image regions related to the image label. We found the effect in images, where pixels next to the actual plant were used for classification.

In contrast to the CNN, where model explanation needs to be done per sample, the unsupervised models were able to extract interpretable image regions. For these models, we were able to statistically select usable features using Spearman’s rank correlation test. We used α=0.1 for this analysis. The classification was done using the remaining features.

Based on our results, we can conclude that the CNN architecture is able to identify complex coherences in image material. Nevertheless, due to the sample-based decision, it is hard to evaluate a general statement for the hidden explanatory factors. In contrast to that, the proposed unsupervised learning models result in saliency maps showing the regions used for classification. We identified these regions as plant as well as background areas. The cAE was able to identify plant specific image regions with few background features. However, the B-GP-LVM extracts plant features with comparatively less information content but was able to identify important background information. In contrast to CNN, all explanatory factors used for classification are known and visualized. Uninformative features could be excluded. In this study, this was done relying on Spearmans rank correlation test.

Nevertheless, the used models relies on user defined hyperparameters. For the cAE, the selection of the architecture and initialization as well as the code size evaluation must be done. The B-GP-LVM is heavily based on the number of features and auxiliary points. However, we were able to implement statistical methods for B-GP-LVM optimization as well as feature selection. Still, the cAE architecture and initialization is an arbitrary choice and cannot be optimized yet. However, the results show that both models were able to solve the classification task.

Relying on two data sets, we found that the explanatory factors of the CNN decision were uninterpretable in certain cases. This is especially important when machine learning/deep learning is used for statistical analysis and novelty detection rather than in-field application implementation. Regardless, our findings show that the implemented CNNs draw conclusions based on biological uninformative image regions. Hereby, the use of unsupervised models result in explainable features in the plant identification approach. These explainable features have a significant relation to the label of the images and are coded in the unsupervised models. This is in contrast do deep learning approaches, where the explainability relies on individual predictions [25,26,45].

We conclude that CNNs must be applied carefully, especially when biological context is important. In contrast to similar plant classification approaches such as [7,9,19,20] our method initially extracts regions of interest learned from the images. These regions were used for classification. Our methodology allows biological interpretation, which was reported to be a key factor for plant recognition [18]. Nevertheless, the presented methodology is restricted to classification. In contrast to that, recent deep learning architectures can be used for advanced applications such as pixel-wise plant segmentation [12]. However, the presented method can be used as an alternative to deep learning classification applications, where explanatory factors are more important than prediction accuracy.

## 5. Materials and Methods

The model evaluation of this study is based on an annotated *Daucus carota* and *Beta vulgaris* image data set. The images were processed using different models. The image data sets as well as the processing pipeline are discussed in the following subsection.

### 5.1. Image Data Sets

The images were generated during in-field experiments for mobile robots on several fields of BonaTerra http://www.bonaterra.at (accessed on 3 December 2021) in the northwest of Marchfeld. The Marchfeld is an area in the northeast of Vienna. The experiments were organized by the IDeAS GmbH and Co KG.

To investigate the effect of complex leaf shape and architecture, we analyzed images of *Daucus carota* as well as *Beta vulgaris*. Plants were extracted and background images (e.g., other plants, soil, stones) were randomly chosen and added to the data set. All samples were extracted using a squared bounding box. We generated 700 plant and soil images for *Daucus carota* and 331 images for *Beta vulgaris*.

Following the recommendations of [13], we converted the RGB images to excess green images, where we converted each image to a gray-scale image using

(1) $$I_{ExG}=2.0\cdot I_{G}-I_{R}-I_{B}.$$

The matrices $I_{\left\{R,G,B\right\}}$ represent the RGB image channels and $I_{ExG}$ are the excess green images. In $I_{ExG}$, green pixels appear to be bright, and soil as well as stones tends to be dark. This conversation is implemented in OpenCV [49].

### 5.2. Data Processing

The selection of the models is motivated by unknown behavior concerning the decision procedure of recent supervised CNN-based models. We hypothesize that high prediction performance of CNNs may be based on image regions confounding with the actual image label. To avoid this problem we followed [29], who reported explainable models based on the unsupervised Gaussian process latent variable model [34]. We compared feature extraction performance of the Gaussian process latent variable model to a recent deep learning based counterpart, namely deep convolutional autoencoder [35]. Both models were used for unsupervised plant feature extraction. These features were visualized and used for classification relying on a support vector machine [36].

To reliably compare the aforementioned classification models, we analyzed the classification applying a five-fold cross validation 10 times relying on reshuffled image data sets. To compare the CNN to the proposed unsupervised models, all experiments were based the same randomized ordering of the images. This subsection describes the used models as well as hyperparameter optimization.

#### 5.2.1. Convolutional Neuronal Network

The default model of this study is the ConvNet D CNN (VGG16) [33]. In this study, we used the VGG16 model, where we added a fully connected layer with 1024 neurons including a ReLu activation function [50], a dropout layer (50% dropout rate), and finally a softmax output layer with 2 neurons. An ImageNet [51] initialization was used. Finally, we used Keras data augmentation with the following parameters: rescale = 1/255, rotation range = 20, width shift range = 0.2, height shift range = 0.2, horizontal flip = True and finally fillmode = ’nearest’.

The categorical cross entropy was used as a loss for optimization. This loss was optimized using a batch size of 8, 50 epochs and Keras RMSProp optimizer [52]. We set the learning rate of the optimizer to $10^{-4}$ and used the default setting for all other parameters. The excess green images were reshaped to [64×64×3] pixel. The implementation is based on Keras [53].

The decision of the classification was analyzed using the layer-wise relevance propagation (LRP) [25,26]. Since LRP results in a saliency maps (heatmaps), we applied a statistical significance test for saliency maps [29] resulting in a pixel-wise *p* value based thresholded heatmap. We used α=0.005 for thresholding.

#### 5.2.2. Gaussian Process Latent Variable Model

In contrast to the single-staged CNN model, we followed the recommendation of [29] and implemented the Bayesian Gaussian process latent variable model [39] relying on radial base function kernel including automatic relevance determination. The B-GP-LVM is an extension of the original GP-LVM formulation [34] using variational inference [54]. The GP-LVM tackles the problem of reducing the *D*-dimensional matrix $Y\in R^{N\times D}$ to $X\in R^{N\times d}$ using the probabilistic model

(2) $$p\left(Y|X,\theta \right)=\prod_{n=1}^{N}p\left(Y\left[n,:\right]|X,\theta \right)=\prod_{n=1}^{N}N\left(Y\left[n,:\right]|0,K_{NN}+\beta^{-1}I_{N}\right).$$

Equation (2) shows a set of *N* Gaussian processes (GPs) mapping from a latent feature space to the original image space relying on the kernel matrix $K_{NN}$. In this study, the radial base function kernel [48] was used. The parameter vector of the model θ is learned from data. In this model, every image $I_{j}$ is represented as a vector ${\vec{i}}_{j}=Y\left[j,:\right]$. The extracted features of $I_{j}$ are represented in the vector ${\vec{x}}_{j}=X\left[j,:\right]$. The GP-LVM uses the factorized prior $p\left(X\right)=\prod_{n=1}^{N}N\left(X\left[j,:\right]|0,I\right)$. The inference problem is solved using the factorized approximation $Q\left(X\right)=\prod_{n=1}^{N}N\left(X\left[j,:\right]|{\vec{\mu }}_{n},S_{n}\right)$ by optimizing [39]

(3) $$F\left(Q\right)=\int Q\left(X\right)\log\left(\begin{array}{c} \frac{p(X)p(X|Y)}{Q(X)} \end{array}\right)dX.$$

Variational inference [54] is used for this approximation. This approximation uses sparsed GP’s [55] relying on a small number of auxiliary points. The expectation of the feature distribution (see Equation (2)) is used as the features for this study.

The aforementioned model is based on the definition of the size of the latent space *d*, as well as the number of auxiliary points used by the sparsed GPs. We estimate both hyperparameters using the following procedure [29]:

1. Initial guess of the latent space: We analyzed the image vector matrix Y using the principal component analysis [48]. The number of principal components explaining 75% of variance were used as an initial guess of *d*, the number of latent plant features. We used sklearn’s principal component analysis implementation [56].
2. Estimation of the auxiliary points: The maximum marginal log likelihood was used to find the optimal number of auxiliary points in a range of {50,75,100,125,150}. We used the minimum number of auxiliary points above 95% of the maximum marginal log likelihood.
3. Latent dimension estimation: The number of latent dimensions was estimated using the aforementioned optimized number of auxiliary points and the maximum marginal log likelihood in a range of {10,25,50,75,100,150} latent dimensions.

The estimated hyperparameters were used to fit a B-GP-LVM. We rely on the GPy [57] implementation using the default parameters.

In addition to the manual feature investigation of [29], we selected features for classification by analyzing the relevance values of the kernel [58] and significance using the *p*-values of Spearman’s rank correlation test.

Initially, we sort the features in accordance to the relevance and calculate the relevance increase per feature. If this value drops below the median of relevance increase, the feature is referred to as being uninformative. Finally, we sort the features in accordance to the relevance and ignore all features after the first uninformative feature occurs.

Afterwards, we implemented a Spearman rank correlation test evaluating $H_{0,j}$: *there is no significant relation between the feature j and the label* for each feature. If the *p* value of a feature is below α=0.1, we can reject the null hypothesis. The *pspearman* [59] package in cran R [60] was used for this test.

**Figure 11:**
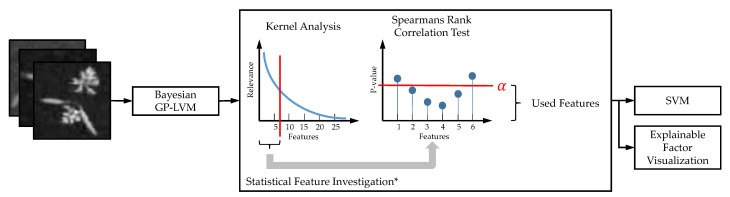
The implemented processing pipeline for B-GP-LVM based feature extraction. We initially use the kernels relevance values for feature pre-selection. Afterwards, we rely on Spearmans rank correlation test to identify the features with significant relation to the label. We extend the pipeline previously published in [29] by our statistical feature selection (see asterisk).

The implemented processing pipeline is visualized in Figure 11.

Different to [29], we applied statistical feature investigation instead of manual feature selection.

Finally, for feature visualization we follow [29,37] and sample from the Gaussian distributed feature space to visualize relevant image regions. To this end, we create for each latent dimension *d* and sample *n* the vector ${}_{n}{\vec{f}}_{d}$. Each entry in this vector is set to the mean of the expectation values of the estimated features. To evaluate the feature dimension *d*, we create *N* vectors and set the *d*-th entry to the actual expectation value of the samples. The set of the created plant feature vector is projected in the image space. The pixel-wise variance of this projection is visualized and interpreted as explanatory factors extracted by the B-GP-LVM. The explanatory factors are visualized in saliency maps. We used a statistical test based on [29] for a pixel-wise and *p* value-based thresholding of the saliency maps to get the significant image regions of interest with α<0.001.

#### 5.2.3. Deep Convolutional Autoencoder

Alternatively to the B-GP-LVM, we used a deep convolutional autoencoder (cAE) [35]. Similarly to the B-GP-LVM, the cAE estimates features representative for the plant image data set. However the cAE uses an encoder/decoder relying on an architecture of artificial neurons to estimate these features. The encoder and decoder are connected through a bottleneck, the so-called code. We use the code as features. While the encoder reduces the image, the decoder recreates the image based on this reduction. The cAE is implemented in Keras [53].

We optimized the cAE using the Keras Adam optimizer, a batch size of 8, and the binary cross entropy as a loss. We used the VGG16 model as an encoder backbone. The encoder was initialized using Imagenet [51]. Since feature dimension optimization similar to B-GP-LVM is not possible, the code size is varied in a range of {10,20,50,75,100} features. The optimal code size was estimated during experiments relying on the aforementioned code sizes in ten iterations. We used the cAE with the lowest mean squared error and applied the Spearmans rank correlation test procedure mentioned above for feature selection.

**Figure 12:**
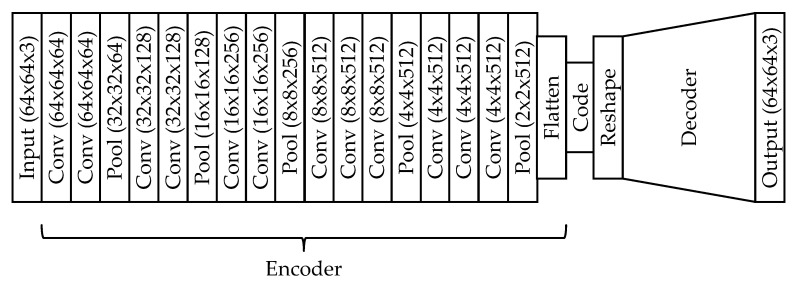
Architecture of the convolutional autoencoder. The VGG16 architecture was used to implement the encoder. The decoder is implemented using the inverted structure of the encoder. We optimized the number of neurons in the code using the mean squared error.

The decoder is based on an inverted architecture of the encoder relying on the ReLu activation function. The implemented architecture is shown in Figure 12.

The visual model investigation is implemented using the sampling procedure of the B-GP-LVMs. We estimated the mean values in the code, and for each dimension in the code, we used the predicted values. The resulting vector is propagated through the decoder. We then analyze the variance resulting in the image space. Again, we used α=0.001 for the statistical analysis of the saliency map.

#### 5.2.4. Statistical Assessment of Saliency Maps

Layer-wise propagation of relevance (LPR) [26] and latent representations of input features such as GP-LVM [34,39,61] and cAE provide metrics which code the importance of input features for the decision making process of machine learning methods. It is common practice in image analysis to visualize LPR, GradCAM, or GP-LVM variance features in so called saliency maps. While providing useful information to identify important image regions, visualizing raw metric values as images puts the burden of interpretation on the user of the algorithms.

To increase the objectivity in deciding the important features [29] proposed a systematic analysis of saliency maps with a significance test. Examining the saliency maps, which are for example found in [21,25,26,29] reveals two common characteristics. Firstly, saliency maps are sparse and secondly important features appear in saliency maps as connected regions with positive metric values. For an objective identification of important regions in saliency maps, we have to formulate this observation quantitatively. The derivation in [29] suggests that features manifest as marked spatial point processes and irrelevant regions may be described by a null hypothesis that metric values are drawn from a marked homogeneous spatial Poisson process. To assess whether a map position belongs to a feature, we compare the null hypothesis against a one sided alternative, which metric values are drawn from a marked generic spatial point process.

These assumptions allow [29] to apply a non-parametric procedure to obtain location specific *p*-values. The saliency map is to this end smoothed by convolving with a k×k dimensional Gaussian kernel to obtain for every location a smoothed saliency map value $s{\left(x,y\right)}_{alt}$. Samples under the null hypothesis are generated by randomizing the locations of saliency map entries and smoothing with the same kernel to obtain $s{\left(x,y\right)}_{null}$. Evidence in favor of the null hypothesis against the one sided alternative is gained if $s{\left(x,y\right)}_{alt}\leq s{\left(x,y\right)}_{null}$. By repeating *N* times randomization, smoothing and comparing values, we obtain, $n{\left(x,y\right)}_{null}$, as the number of instances in support of the null hypothesis. With *N* chosen suitably large provides location specific *p*-values $p_{sm}\left(x,y\right)$. Thresholding $p_{sm}\left(x,y\right)$ with a suitably small value ($p_{thrs}=\alpha$) provides the locations of important saliency map features in a reproducible manner.

We use $N=10^{4}$, k=5 and α=0.001 in this study. The test is implemented in OpenCV [49] using Python.

#### 5.2.5. Classification of Latent Features

The estimated features of the cAE as well as the B-GP-LVM are used for classification. The classification is implemented using a ν support vector machine and the rbf kernel [36]. Similar to the CNN pipeline, we use 10 five-fold cross validation iteration relying on reshuffled data sets. The hyperparameters of the kernel function and the SVM are optimized using global Bayesian optimization [47] relying on ten initialization and ten optimization points. This optimization is implemented using [62]. The experiments are based on the e1071 R SVM implementation [63] and use the logistic regression-based extension to obtain prediction probabilities.

## 6. Conclusions

We hypothesized in this study that state-of-the art classification models based on convolutional neuronal networks may classify images based on features that cannot be interpreted in a biological way. Further, we hypothesized that unsupervised learning based methodologies perform similarly but overcome the black-box model problem. Both hypotheses were evaluated based on two plant classification data sets. We used a *Daucus carota* and a *Beta vulgaris* image data set to evaluate different leaf shape and leaf architecture complexity. In our experiments, we analyzed the prediction accuracy and mutual information. Additionally, the McNemar test was used to test the prediction performance differences of the proposed unsupervised models to a state-of-the-art CNN.

For both data sets, we found image regions that cannot be explained in a biological way that were used by the CNN in the best iteration and best fold. We extracted and visualized features using unsupervised machine learning models. Those models were implemented relying on the Bayesian Gaussian process latent variable model and deep convolutional autoencoders. The features were visualized using the methodology of [29]. The explanatory factors used for classification were visualized. In contrast to the CNN, where the explanatory factors are latent in the learned model, all classification decisions of the unsupervised learning-based approaches are based on those features.

Finally, we analyzed the prediction performance of the used classifiers. The CNN outperforms the other approaches in terms of prediction accuracy and mutual information. Nevertheless, the McNemar test showed insignificant performance differences for the *Beta vulgaris* data set.

We conclude that CNNs must be applied carefully. We found that unsupervised learning based methodologies, where all used image regions can be visualized before classification, are able to solve the classification task but do perform inferior regarding the prediction accuracy. Nevertheless, we can be sure that the unsupervised models do not use any features that cannot be explained.

Our next steps includes the integration of extensions of the B-GP-LVM (e.g., deep Gaussian processes [64]) as well as the implementation of recent CNN backbones for the cAE architecture (e.g., ResNet [10]). Finally, we will combine the features of the Bayesian Gaussian process latent variable model and the cAE. 

## Figures and Tables

**Table 1:** Summarized explanation methods for machine learning based computer vision models.

Method	Models	Explanation Strategy
Eigenfaces [41]	PCA	Model parameter investigation
LRP [21,25,26], SpRAy [27,46]	Deep CNNs	Post hoc CNN prediction analysis
Visualization for B-GP-LVM [29]	GP-LVM	Model investigation
Our contribution	GP-LVM and deep cAE	Model investigation & feature selection

## Data Availability

The used data sets as well as the implemented software is available at https://github.com/TW-Robotics/Plant_Unsupervised, accessed on 3 December 2021.

## Abbreviations

The following abbreviations are used in this manuscript:

B-GP-LVM	Bayesian Gaussian process latent variable model
cAE	(Deep) convolutional autoencoder
CNN	Convolutional neuronal network
GP-LVM	Gaussian process latent variable model
LRP	Layer-wise relevance propagation
SVM	Support Vector machine
